# Genome Sequences of Sequence Type 45 (ST45) Persistent Methicillin-Resistant *Staphylococcus aureus* (MRSA) Bacteremia Strain 300-169 and ST45 Resolving MRSA Bacteremia Strain 301-188

**DOI:** 10.1128/genomeA.00174-14

**Published:** 2014-03-13

**Authors:** David Hernandez, Kati Seidl, Anna-Rita Corvaglia, Arnold S. Bayer, Yan Q. Xiong, Patrice François

**Affiliations:** a Genomic Research Laboratory, University of Geneva Hospitals, Geneva, Switzerland; b University Hospital Zurich, University of Zurich, Zurich, Switzerland; c Los Angeles Biomedical Research Institute at Harbor-UCLA Medical Center, Torrance, California, USA; d David Geffen School of Medicine at UCLA, Los Angeles, California, USA

## Abstract

Persistent methicillin-resistant *Staphylococcus aureus* (MRSA) bacteremia (positive blood cultures after ≥7 days) represents a challenging subset of invasive MRSA infections. The comparison of genome sequences of persistent (300-169) and resolving (301-188) MRSA bacteremia isolates with similar genetic background (sequence type 45 [ST45]) will help us to better understand underlying mechanisms of persistent MRSA bacteremia.

## GENOME ANNOUNCEMENT

Persistent methicillin-resistant *Staphylococcus aureus* (MRSA) bacteremia (PB) (positive blood cultures after ≥7 days) comprises 20 to 30% of all episodes of MRSA bacteremia and is especially relevant to endovascular infections (1). The reasons why some MRSA bacteremia strains persist while others resolve during antimicrobial therapy despite similar clinical and microbiologic characteristics are not well understood. Here we report the complete genome sequences of PB (strain 300-169) and resolving MRSA bacteremia (RB; negative blood cultures after 2 to 4 days of therapy) (strain 301-188) clinical isolates with similar genetic background (sequence type 45 [ST45]) which originated from a multinational *S. aureus* bacteremia clinical trial collection (2). While strain 300-169 was isolated from a patient with 16 days of MRSA bacteremia, strain 301-188 was from a patient with 2 days of MRSA bacteremia. The two strains were tested for several phenotypes that are thought to influence clinical outcomes. Strain 300-169 exhibited significantly better survival in the presence of host defense cationic peptides, formed significantly more biofilm, and induced significantly more endothelial cell damage than strain 301-188. Both strains were similar in terms of virulence in a rabbit infective endocarditis model, but in contrast to strain 301-188, strain 300-169 was resistant to vancomycin therapy even though both strains are susceptible to vancomycin *in vitro* (MIC, 0.5 µg/ml for both strains) (3–6).

Purified genomic DNA was subjected to whole-genome shotgun sequencing by using a HiSeq system (Illumina, Inc.). Following fragmentation, end reparation, and sample tagging, the sequencer produced 32.4 and 32.9 million paired reads for strains 300-169 and 301-188, respectively, yielding appreciable coverage of ~1,000× for both strains. Assembly was performed using Edena v. 140122 (7). Strain 300-169 assembly resulted in 37 contigs (sum, 2.81 Mbp; *N*_50_, 469 kbp; maximum length, 1.0 Mbp), while strain 301-188 assembly resulted in 40 contigs (sum, 2.76 Mbp; *N*_50_, 412 kbp; maximum length, 598 Kbp). Annotations were performed by the NCBI Prokaryotic Genomes Annotation Pipeline. The chromosome of strain 300-169 contains 2,620 coding sequences (CDSs), 22 rRNAs, and 60 tRNAs, while the chromosome of strain 301-188 contains 2,539 CDSs, 25 rRNAs, and 60 tRNAs. For each strain, >52% of the genes were assigned to specific subsystem categories by RAST (7). Comparison of the two genomes revealed 137 single nucleotide polymorphisms (SNPs).

Strain 300-169 contains one circular plasmid of 2.6 kb at an apparent copy number of 25. Interestingly, 300-169 has a specific bacteriophage of 44.2 kb and a highly mosaic prophage showing partial homology with phi11, phi69 on the 5′ extremity, and phiSLT on the 3′ end. Phages have been shown to play a role in *S. aureus* pathogenicity by encoding diverse virulence factors or by insertion into and thereby interruption of chromosomal *S. aureus* virulence genes (i.e., β-hemolysin [*hlb*] and lipase [*geh*] genes) (8). The new phage in strain 300-169 is inserted in a metabolic gene with hypothetical function involved in oxidoreduction reactions. Importantly, phages can also influence genome plasticity and thus adaptation to the host (8, 9). Studies to further examine the gene contents and prophages that differ between the two isolates and their roles in persistent MRSA bacteremia are in progress in our laboratories.

### Nucleotide sequence accession numbers.

The whole-genome sequences of MRSA 300-169 and 301-188 were deposited in the DDBJ/EMBL/GenBank databases under the accession numbers JASL00000000 and JASK00000000, respectively.
